# Room-temperature crystal structure of a metal-dependent W-formate dehydrogenase by serial synchrotron crystallography

**DOI:** 10.1107/S2059798326006923

**Published:** 2026-08-10

**Authors:** Guilherme Vilela-Alves, Guilherme Martins, David von Stetten, Pedram Mehrabi, Inês A. C. Pereira, Maria João Romão, Arwen R. Pearson, Cristiano Mota

**Affiliations:** a Associate Laboratory i4HB – Institute for Health and Bioeconomy and UCIBIO, Applied Molecular Biosciences Unit, Department of Chemistry, NOVA School of Science and Technology Universidade Nova de Lisboa 2829-516Caparica Portugal; b Instituto de Tecnologia Química e Biológica António Xavier Universidade Nova de Lisboa Avenida da República 2780-157Oeiras Portugal; c European Molecular Biology Laboratory (EMBL) Hamburg Unit c/o DESY, Notkestrasse 85 22607Hamburg Germany; d Institute for Nanostructure and Solid-State Physics Universität Hamburg HARBOR, Luruper Chaussee 149 22761Hamburg Germany; Institute of Integrative Biology, University of Liverpool, United Kingdom

**Keywords:** CO_2_ reduction, metal-dependent formate dehydrogenases, Mo/W enzymes, serial synchrotron X-ray crystallography, room-temperature X-ray crystallography

## Abstract

Metal-dependent formate dehydrogenases (Fdhs) are a promising research target in efforts to mitigate climate change by developing active, efficient, selective and safe industrial catalysts for CO_2_ reduction. Here, we report the room-temperature (RT) serial synchrotron X-ray crystallography (SSX) structure of *Nitratidesulfovibrio vulgaris* formate dehydrogenase AB and compare this structure with previously reported cryogenic structures. The comparison reveals structural differences likely arising from the absence of cryoprotectants, differences in data-collection temperature and the much lower radiation dose accumulated by each crystal during the SSX experiments. The RT-SSX structure provides the first step towards time-resolved serial crystallography experiments targeting possible catalytic intermediates in order to fully understand the catalytic mechanism of metal-dependent Fdhs.

## Abstract 2

We report a room-temperature serial synchrotron X-ray crystallographic structural analysis of *Nitratidesulfovibrio vulgaris* formate dehydrogenase AB, an enzyme that reduces CO_2_ to formate.

## 1. Introduction

Efficient strategies for converting carbon dioxide (CO_2_) into value-added products are in high demand to mitigate climate change but remain limited due to the thermodynamically stable nature of CO_2_, which makes these reactions chemically challenging and difficult to implement industrially (Appel *et al.*, 2013 ▸; Calzadiaz-Ramirez & Meyer, 2022 ▸; Aresta *et al.*, 2013 ▸). However, nature has evolved a highly efficient and selective solution in formate dehydrogenases, and there is significant interest in developing optimized enzymes and bio-inspired synthetic catalysts for large-scale industrial use (Szczesny *et al.*, 2020 ▸; Lodh & Roy, 2022 ▸; Kanega *et al.*, 2020 ▸). Metal-dependent (molybdenum/tungsten) formate dehydro­genases (Fdhs) are a structurally modular family of enzymes. They contain a common conserved catalytic subunit and diverse additional subunits (containing different cofactors ranging from iron–sulfur clusters to haem groups and flavins). This allows cells to integrate these enzymes into multiple metabolic pathways (da Silva *et al.*, 2013 ▸) where they can interface with different intracellular electron carriers (Boyington *et al.*, 1997 ▸; Jormakka *et al.*, 2002 ▸; Radon *et al.*, 2020 ▸; Young *et al.*, 2020 ▸; Oliveira *et al.*, 2020 ▸; Raaijmakers *et al.*, 2002 ▸). The conserved catalytic subunit catalyses the reversible conversion of CO_2_ to formate and features a Mo/W active site that is responsible for its significantly higher turnover rates compared with the structurally distinct metal-independent Fdhs (Maia *et al.*, 2015 ▸; Nielsen *et al.*, 2019 ▸). The active site presents a distorted trigonal prismatic geometry, in which the metal ion (Mo or W) is hexacoordinated to a (Se)Cys residue from the peptide chain, a terminal sulfido (–SH/=S) ligand and two dithiolene groups from two molybdo­pterin guanine dinucleotides (MGDs) (Romão, 2009 ▸; Hille *et al.*, 2014 ▸; Grimaldi *et al.*, 2013 ▸), one of which is responsible for electron transfer (MGD1; Oliveira *et al.*, 2025 ▸; Raaijmakers *et al.*, 2002 ▸). The catalytic site is completed in the second coordination sphere by a highly conserved histidine residue with an assigned putative proton-transfer role, and a conserved arginine residue, likely involved in substrate and catalytic intermediate orientation (Oliveira *et al.*, 2020 ▸; Siegbahn, 2022 ▸; Hartmann *et al.*, 2016 ▸; Dong & Ryde, 2018 ▸; Axley *et al.*, 1990 ▸). The heterodimeric W-FdhAB from *Nitratidesulfovibrio vulgaris* (*Nv*), a SeCys-containing enzyme with four [4Fe–4S] clusters (Oliveira *et al.*, 2020 ▸), has been extensively studied due to its high activity and oxygen tolerance (Oliveira *et al.*, 2024 ▸). However, to date all structural studies reported were conducted under cryo conditions (100 K; Oliveira *et al.*, 2020 ▸; Vilela-Alves *et al.*, 2023 ▸).

Previous studies have shown that upon reduction (with formate or dithionite) the catalytic amino acids Arg441 and His193 adopt different conformations, along with structural changes in the Sec192–Thr196 helix, the MGD2 cofactor and the neighbouring residues Glu443 and Gln890 (Oliveira *et al.*, 2020 ▸). Additionally, a disulfide bond-based activation mechanism was also uncovered, using the C872A variant as a proxy for the open conformation of the surface-exposed Cys845–Cys872 disulfide (S—S) bond. The activation/deactivation stimulus was found to be conveyed from the surface to the buried active site (25 Å away) through a complex allosteric mechanism featuring, in the vicinity of the active site, the residues Gln409 and Met405, which in turn impact the MGD2 and the W site (Oliveira *et al.*, 2024 ▸). Lastly, a substrate-retention site, comprising His193, Val197, Arg441, Gln447 and Thr450, was identified in the main substrate tunnel of *Nv*FdhAB, very close to the active site. This retention site plays a synergistic role by increasing substrate concentration near the W site, thereby enhancing CO_2_ reduction and improving catalytic efficiency (Vilela-Alves *et al.*, 2026 ▸).

Here, we report the crystal structure of the *N. vulgaris* metal-dependent formate dehydrogenase AB (*Nv*FdhAB) obtained by room-temperature (RT) serial synchrotron X-ray crystallography (SSX). A comparison with the previous cryo crystallographic structures reveals conformational changes in key residues and in the hydration water network that likely arise from the difference in data-collection temperature and the absence of cryoprotectant molecules. Leveraging the low radiation dose achievable in SSX, the geometry of the W active site could be used to ascertain the impact of the putative photoreduction of the W ion on the active-site structure in previous high-dose structures (for example PDB entry 6sdr; Oliveira *et al.*, 2025 ▸). Given the successful optimization of *Nv*FdhAB microcrystals and of SSX data collection, our results pave the way for future time-resolved SSX experiments, aiming to better probe the still intriguing catalytic mechanism for CO_2_ reduction.

## 2. Materials and methods

### 2.1. Expression and purification of *N. vulgaris* FdhAB

*Nv*FdhAB was overexpressed and affinity-purified from *N. vulgaris* Hildenborough as described previously (Oliveira *et al.*, 2020 ▸; Supplementary Table S1). Protein concentration was determined using ɛ_410 nm_ = 43.45 m*M*^−1^ cm^−1^. To verify batch quality, denaturing acrylamide gel electrophoresis and kinetic assays were performed as reported in Oliveira *et al.* (2020 ▸).

### 2.2. Crystallization, data collection, structure solution and refinement

*Nv*FdhAB at 10 mg ml^−1^ was used to produce microcrystals through batch crystallization in 500 µl microtubes at 20°C in 30 µl batches containing precipitant solution consisting of 32%(*w*/*v*) PEG 3350, 0.1 *M* Tris–HCl pH 8.0, 1 *M* LiCl (Oliveira *et al.*, 2020 ▸) and microseeds of wild-type (WT) *Nv*FdhAB, with a ratio of 15:10.5:4.5 µl protein:precipitant:microseeds (Supplementary Table S2). Microseeds were obtained by harvesting macrocrystals of WT *Nv*FdhAB into 50 µl reservoir solution and vortexing them with a plastic bead ten times for 30 s on and 30 s off. To obtain *Nv*FdhAB macrocrystals, the hanging-drop vapour-diffusion method was used at 20°C in 24-well plates (24-well XRL plate, Molecular Dimensions) containing 2 µl drops (1:1 protein:precipitant ratio). WT *Nv*FdhAB (10 mg ml^−1^) was crystallized using precipitant solutions consisting of 22–26%(*w*/*v*) PEG 3350, 0.1 *M* Tris–HCl pH 8.0, 1 *M* LiCl (Oliveira *et al.*, 2020 ▸) supplemented with 0.2 µl of a 1:500 dilution from a stock of microseeds of WT *Nv*FdhAB. Both macro and micro crystals first appeared after 24 h and were harvested after 72 h.

The microcrystal slurry was then loaded onto previously glow-discharged HARE chips (featuring inverted frustum-shaped crystal cavities with a base edge of 10 µm, a top edge of 82 µm and a height of 50 µm; manufactured by the Max Planck Semiconductor Laboratory, Garching, Germany), following the protocol developed by Mehrabi *et al.* (2020 ▸). Briefly, 90 µl of microcrystal slurry was loaded onto a HARE chip inside a humidity-controlled hood (relative humidity 95%), followed by the application of a gentle vacuum to the back of the chip to remove excess mother liquor before the HARE chip was mounted in a specialized chip holder. Data collection was conducted on EMBL beamline P14-2 (T-REXX; Horrell *et al.*, 2019 ▸) at the PETRA III synchrotron, DESY, Hamburg, Germany with the HARE chip placed inside a temperature- and humidity-controlled chamber (temperature 20°C, relative humidity 95%), using the previously described translation-stage holders (Mehrabi *et al.*, 2020 ▸; Schulz *et al.*, 2025 ▸). The exposure time per crystal was 5 ms, and the beam flux apertured to 10 × 7 µm (horizontal × vertical) was 1.1 × 10^12^ photons s^−1^ in a 30 × 7 µm (horizontal × vertical) Gaussian beam. Three full HARE chips were measured and the data obtained from the three chips were merged into a single dataset, with a moderately low average hit rate of 18% and a total of 11 144 indexed lattices.

Macrocrystals (thin plates of approximately 300 × 200 × 30 µm) were harvested and then flash-cooled in liquid nitrogen using the precipitant solution supplemented with 20%(*v*/*v*) glycerol as a cryoprotectant. X-ray diffraction experiments on the macrocrystals under cryogenic conditions (100 K) were performed on EMBL beamline P14 at the PETRA III synchrotron, DESY, Hamburg, Germany.

SSX data were processed with *CrystFEL* v0.10.2 (White *et al.*, 2012 ▸) using the *XGANDALF* indexer (Gevorkov *et al.*, 2019 ▸) and *partialator* for scaling and merging with the unity partiality model, while macrocrystal cryo data were processed with *autoPROC* (Vonrhein *et al.*, 2011 ▸). For both datasets, *Phaser* 2.8.3 (McCoy *et al.*, 2007 ▸) from the *CCP*4 suite 7.1.018 (Agirre *et al.*, 2023 ▸) was used to solve the structure by molecular replacement, using the WT *Nv*FdhAB oxidized structure (PDB entry 6sdr; Oliveira *et al.*, 2025 ▸) as a search model. Cycles of automatic restrained refinement using *REFMAC* 5.8.0267 (Murshudov *et al.*, 2011 ▸) and manual model building using *Coot* 0.8.9.1 (Emsley *et al.*, 2010 ▸) were conducted to iteratively refine the model. After initial refinement, the online *PDB-REDO* server (Joosten *et al.*, 2014 ▸) was used to rebuild the model. Publication images were generated in *PyMOL* 4.6.0 (Schrödinger). Statistics on data collection and processing and on model refinement are presented in Table 1 ▸. Average dose for each dataset was estimated using the *RADDOSE*-3*D* online server (input data available in Supplementary Data S1; Dickerson *et al.*, 2024 ▸).

## 3. Results and discussion

### 3.1. Room-temperature synchrotron serial crystallographic structure of *Nv*FdhAB

**Figure 1:**
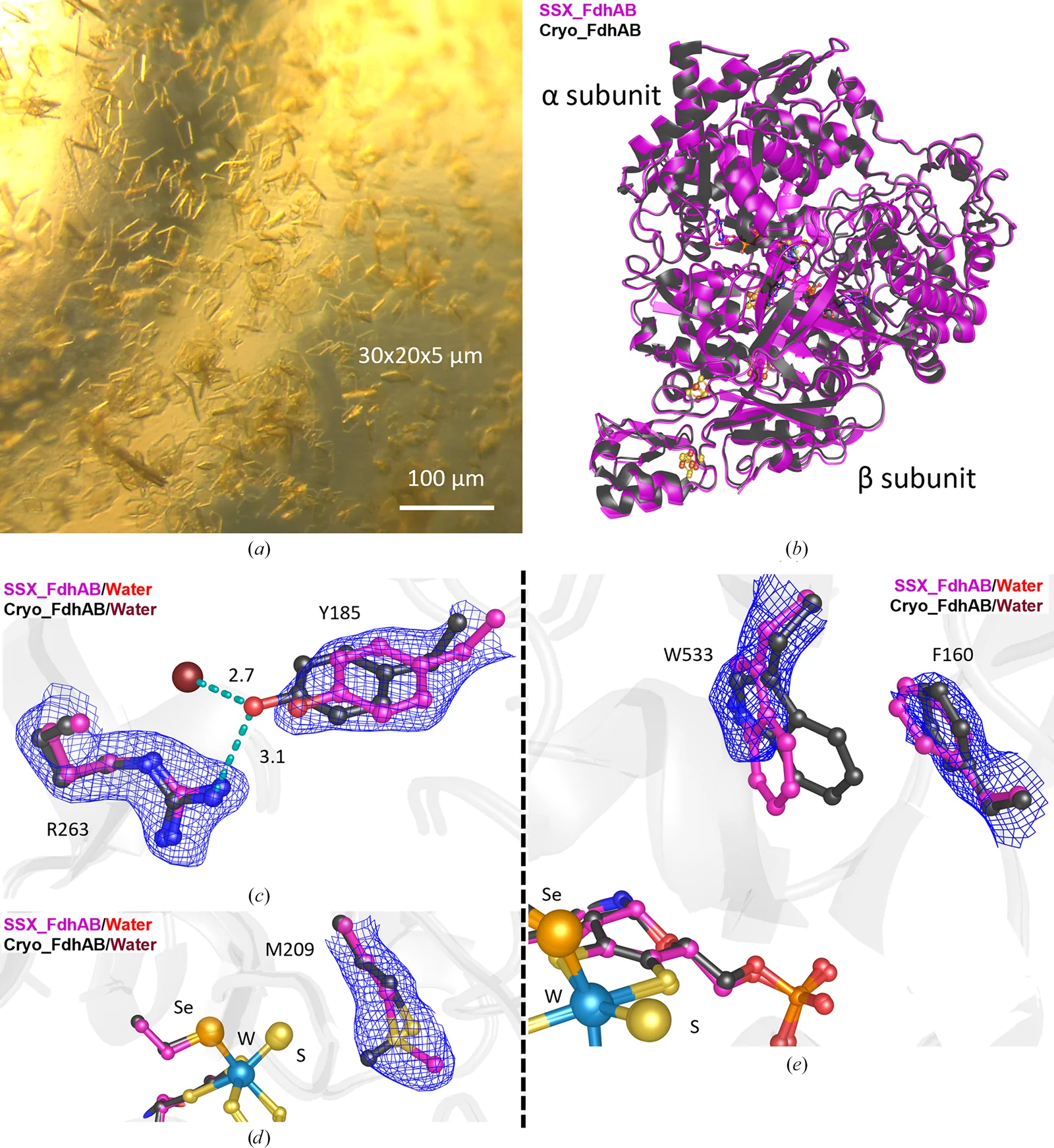
(*a*) Microphotograph of *Nv*FdhAB microcrystals, approximately 30 × 20 × 5 µm in size, in suspension in mother liquor. (*b*) Superposition of the RT-SSX and cryo *Nv*FdhAB (PDB entry 6sdr) structures in violet and black, respectively. The W site with the two MGD cofactors and the four [4Fe–4S] clusters shown in ball-and-stick representation. (*c*, *d*, *e*) Examples of the differences in side-chain positioning and hydration water location between the superimposed SSX and cryo *Nv*FdhAB (PDB entry 6sdr) structures in violet and black, respectively, around residues Tyr185 and Arg263, Phe160 and Trp533 and, lastly, Met209. In (*c*) the absent water molecule is shown as a burgundy sphere. Hydrogen bonds are shown as teal dashes and distances are in Å. RT-SSX 2*mF*_o_ − *DF*_c_ electron-density maps are shown as a blue mesh at 1.0 r.m.s.d.

*Nv*FdhAB in the aerobically as-isolated state (Oliveira *et al.*, 2020 ▸) yielded microcrystals as moderately homogeneous thin plates of approximately 30 × 20 × 5 µm (Fig. 1 ▸*a*) belonging to space group *P*2_1_2_1_2_1_ that diffracted to 1.93 Å resolution (Table 1 ▸). It is interesting to note a slight increase in unit-cell dimensions between the room-temperature and cryo structures, with the maximum difference (2.5 Å, approximately 1.6%) in the *c* axis (the unit-cell histogram for the serial dataset is shown in Supplementary Fig. S1), which is an expected temperature effect. Data-processing statistics indicate 100% completeness and the model was refined with good geometry indicators (*MolProbity* score of 1.17), with *R*_work_ and *R*_free _values of 0.185 and 0.222, respectively (Table 1 ▸). As expected, the overall RT-SSX *Nv*FdhAB structure is similar to the cryo structure (Fig. 1 ▸*b*). Superposition of the SSX structure with the cryo structure (PDB entry 6sdr) yields an overall r.m.s.d. of 0.39 Å for 1178 C^α^ atoms and r.m.s.d.s of 0.35 Å for 963 C^α^ atoms and 0.31 Å for 215 C^α^ atoms for chains *A* and *B*, respectively. A higher overall r.m.s.d. value reflects a slight change in the relative orientation of the two subunits. However, this variation falls within the range observed among previously reported cryo structures. Nonetheless, the room-temperature data revealed subtle structural changes in side-chain positioning and hydration water location (examples are shown in Figs. 1 ▸*c*, 1 ▸*d* and 1 ▸*e*). To ensure that enough diffraction patterns were collected to yield good-quality data, we deleted Trp728 from the model and the structure was re-refined. The fact that the proper tryptophan-shaped electron density remains in the omit map (Supplementary Fig. S2) confirms that data of sufficient quality have been collected (von Stetten & Pearson, 2026 ▸).

### 3.2. Structural comparison of *Nv*FdhAB from serial and cryo crystallography

**Figure 2:**
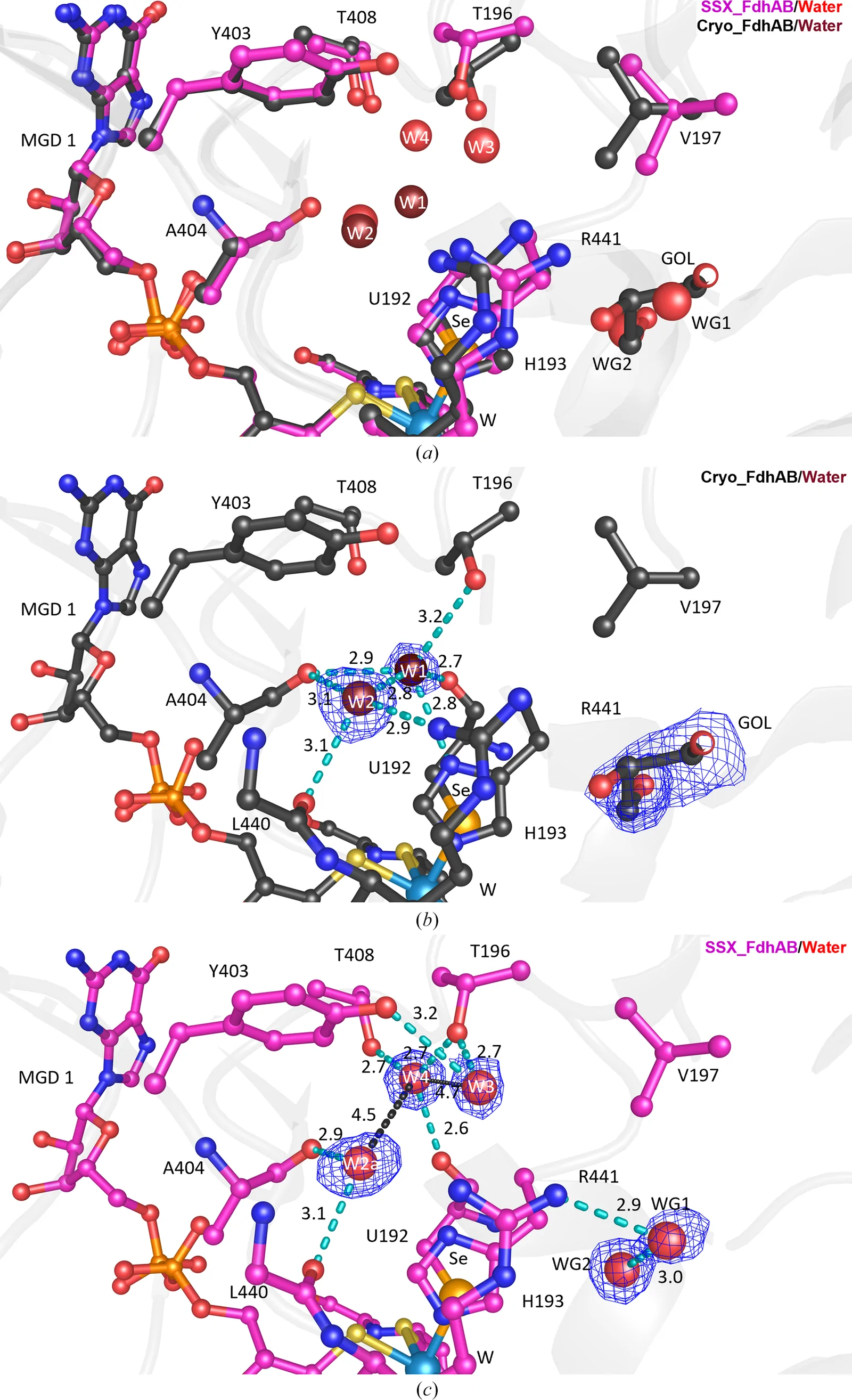
Differences in the vicinity of the retention site (Vilela-Alves *et al.*, 2026 ▸) between the SSX and cryo *Nv*FdhAB (PDB entry 6sdr) structures in violet and black, respectively. In all representations, the W active site is shown in ball-and-stick representation; the W ion, sulfido group and Sec192 Se atom are shown as spheres (light blue, yellow and orange, respectively). 2*mF*_o_ − *DF*_c_ electron-density maps are shown as a blue mesh at 1.0 r.m.s.d. Hydrogen bonds and distances are shown as teal and black dashes, respectively. Distances are in Å. (*a*) Superposition of the SSX and cryo *Nv*FdhAB (PDB entry 6sdr) structures. The seven water molecules are shown as spheres in red and burgundy, respectively, for the SSX and cryo structures. (*b*) The cryo *Nv*FdhAB structure (PDB entry 6sdr). (*c*) The SSX *Nv*FdhAB structure.

Since SSX data collection was performed at room temperature, one of the most obvious differences in the structure refined from the SSX dataset compared with the structure determined at 100 K (PDB entry 6sdr) is that no cryoprotectant was required and thus the structurally conserved glycerol molecule, originating from the cryoprotectant and located at the proposed retention site (Vilela-Alves *et al.*, 2026 ▸), was absent. Despite the lack of glycerol binding, only small changes in the positioning of side chains forming this pocket were observed, likely because the site is now occupied by two water molecules, WG1 and WG2 (Fig. 2 ▸). The guanidinium group of the catalytic Arg441 moves 1.0 Å towards the position usually occupied by the glycerol molecule (Fig. 2 ▸*a*) and concomitantly Thr196 and Val197 slightly rearrange. This limited mobility of the residues lining the retention site supports its assignment as a pre-binding site, acting to concentrate substrates, particularly CO_2_, near the W site to enhance catalysis (Vilela-Alves *et al.*, 2026 ▸). This flexibility likely enables optimal transient interactions with the different substrates as they diffuse into the active site (Vilela-Alves *et al.*, 2023 ▸, 2026 ▸).

Moreover, in the cryo structure (PDB entry 6sdr) water W1 is stabilized by hydrogen bonds to the N^δ^ atom of His193 (at 2.9 Å), the carbonyl groups of Ala404 (at 2.9 Å) and Sec192 (at 2.8 Å), the O^γ^ atom of Thr196 (at 3.2 Å) and another water, W2 (at 2.8 Å) (Fig. 2 ▸*b*). W2 is coordinated by the backbone carbonyl of Ala404, the side chain of Arg441, the backbone carbonyl of Leu440 and by W1. In the SSX structure (Fig. 2 ▸*c*), three water molecules are observed in this pocket. W1 is missing and W2a is very close to the position of W2 in the cryo structure but is no longer coordinated by Arg441. Two new water molecules, W3 and W4, are found towards the ‘top’ of this pocket. W3 is hydrogen-bonded to the O^γ^ atom of Thr196 (at 2.7 Å) and the O^η^ atom of Tyr403 (at 3.2 Å). W4 interacts with the carbonyl group of Sec192 (at 2.6 Å), the O^γ^ atom of Thr196 (at 2.7 Å) and Thr408 (at 2.7 Å). Despite the water rearrangements in the SSX structure, all interactions between water molecules and the peptide chain in this pocket observed in the cryo structure are retained, except for the carbonyl group of Ala404, which now only interacts with one water (W2a at 2.9 Å) instead of two. In a broader analysis, it is interesting to note a decrease in the total number of modelled waters in the SSX structure when compared with the cryogenic structures (Fdh_CryoSamePrep and PDB entry 6sdr). A comparison of the two structures shows that most of the missing water molecules are those at the surface of the cryo structure, which indicates that solvent-exposed water molecules are significantly more mobile at room temperature than at 100 K, the latter resulting in a more ordered water network that can be seen in the electron-density maps.

**Figure 3:**
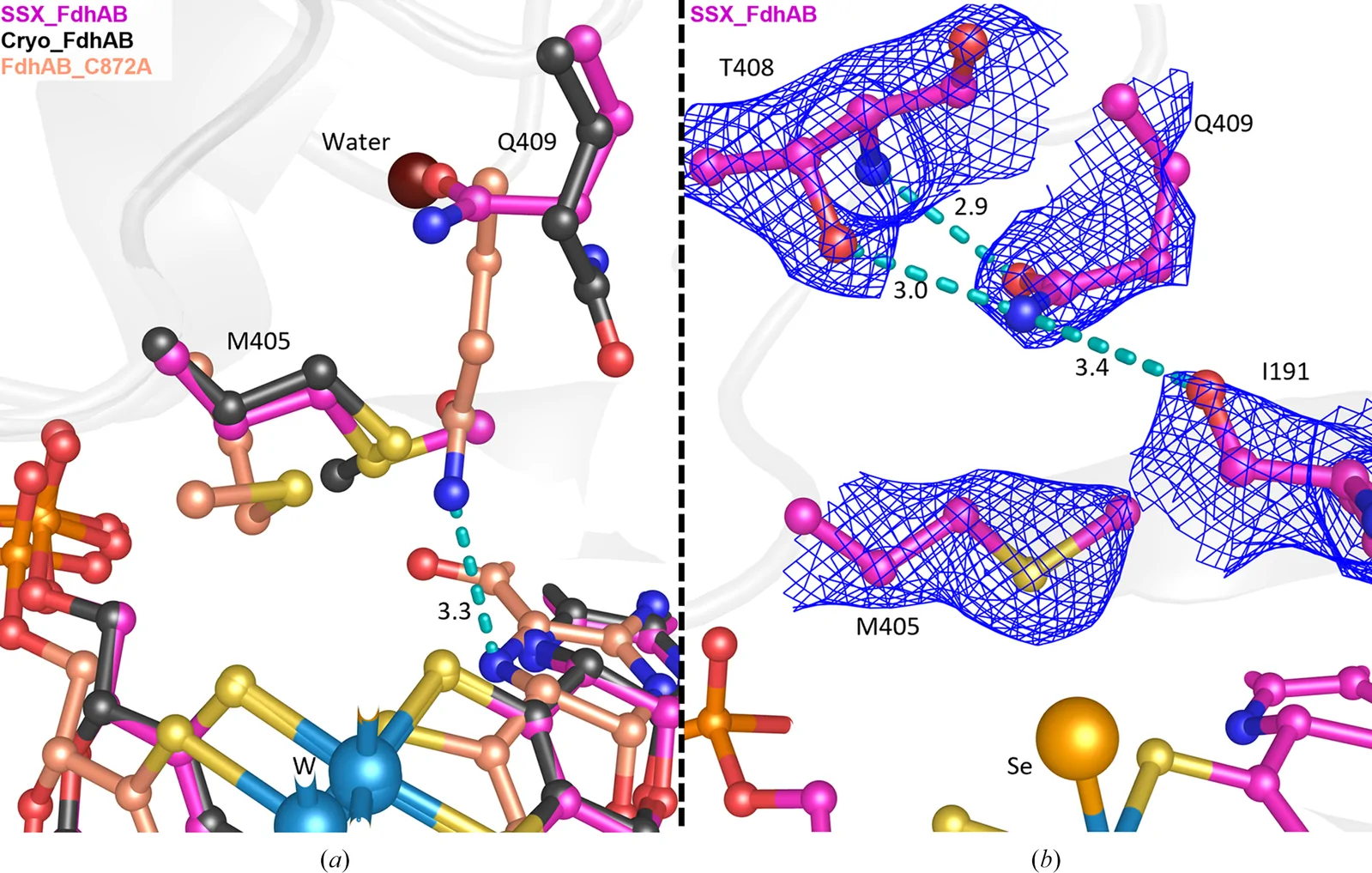
The differences in the vicinity of the MGD2 cofactor between the SSX, cryo (PDB entry 6sdr) and C872A variant (PDB entry 8cm6) *Nv*FdhAB structures in violet, black and orange, respectively, focusing on the key Gln409 and Met405 residues in the cascade of conformational changes that convey the activation/O_2_-protection signal from the surface redox switch to the buried W active site. In all representations, the W active site is shown in ball-and-stick representation; the W ion, sulfido group and Sec192 Se atom are shown as spheres (light blue, yellow and orange. respectively). Hydrogen bonds are shown as teal dashed lines. Distances are in Å. (*a*) Superposition of the SSX, cryo (PDB entry 6sdr) and C872A (PDB entry 8cm6) *Nv*FdhAB structures. The water molecule in the cryo structure is shown as a burgundy sphere. (*b*) Reoriented view of the SSX *Nv*FdhAB structure, highlighting the hydrogen-bonding interactions of Gln409. 2*mF_o_* − *DF*_c_ electron-density maps are shown as a blue mesh at 1.0 r.m.s.d.

Gln409 and Met405 are key residues in the cascade of conformational changes that convey the activation/O_2_-protection signal from the solvent-exposed redox switch (Cys845–Cys872 disulfide bond; Oliveira *et al.*, 2024 ▸) to the buried W active site. In the SSX structure, Gln409 adopts a new rotamer, facing away from the MGD2 pterin moiety of the active site. This conformation displaces a water molecule present in the cryo structure (PDB entry 6sdr) and is stabilized by hydrogen bonds between Gln409 O^ɛ^ and the backbone amine of Thr408 (at 2.9 Å), between Gln409 N^ɛ^ and Thr408 O^γ^ (at 3.0 Å), and a weaker hydrogen bond (at 3.4 Å) between Gln409 N^ɛ^ and the carbonyl group of Ile191 (Fig. 3 ▸). The positioning of Gln409 is closely related to the change in conformation of Met405 (Oliveira *et al.*, 2024 ▸, 2025 ▸; Vilela-Alves, Manuel, Pedrosa *et al.*, 2024 ▸; Vilela-Alves, Manuel, Viegas *et al.*, 2024 ▸), and in the SSX structure Met405 also adopts a different rotamer at the terminal methyl group, facing the vacant position of Gln409 in the cryo structure. These rearrangements are notable as the Cys845–Cys872 disulfide bond is clearly intact in the SSX structure (Supplementary Fig. S3). However, these new conformations do not reflect the Gln409/Met405 conformations observed in the cryo structure of the activated form of the enzyme (open disulfide bond; PDB entry 8cm6; Oliveira *et al.*, 2024 ▸), where Gln409 has undergone a considerable shift to hydrogen-bond to the N5 of MGD2 (Fig. 3 ▸*a*).

**Figure 4:**
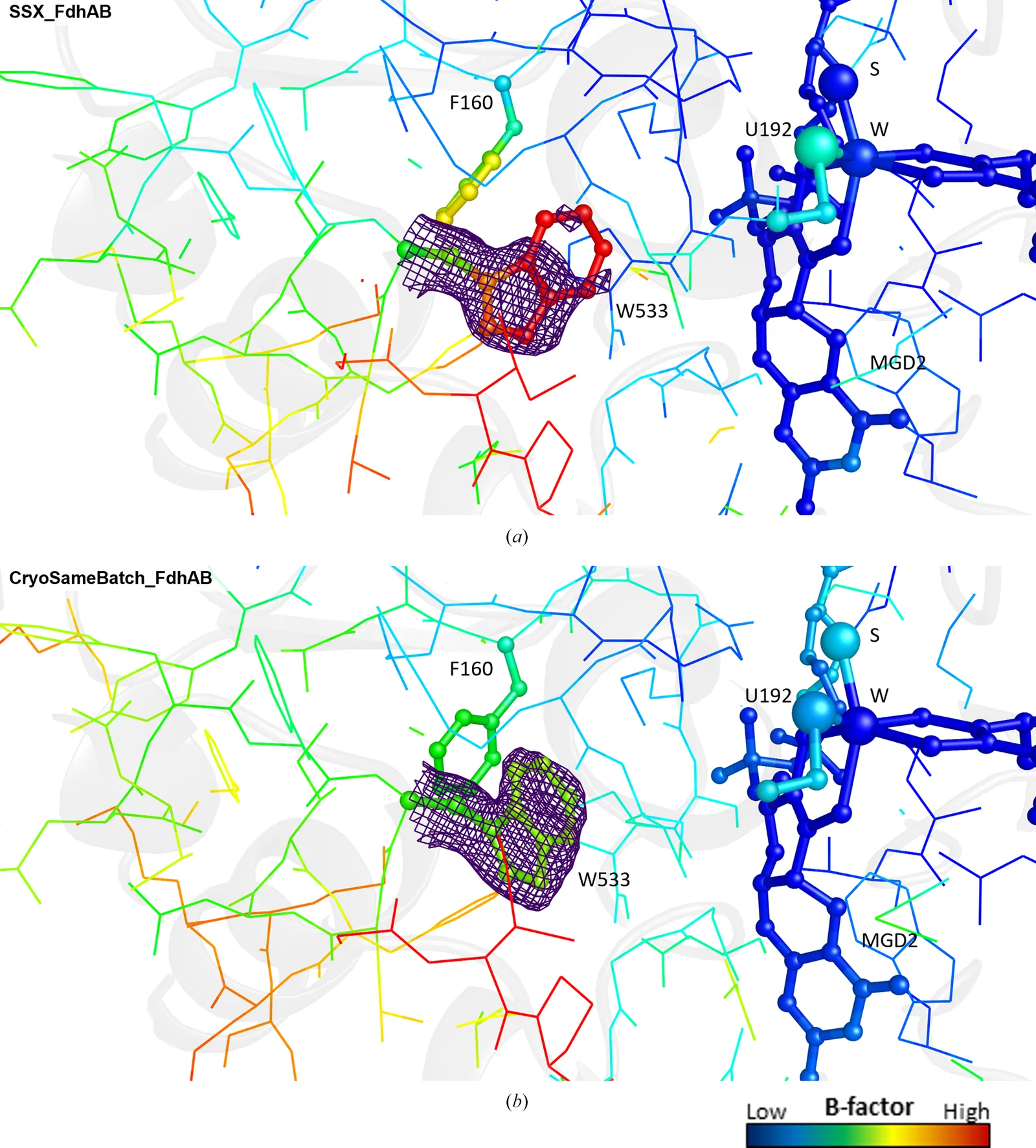
Effect of the data-collection temperature on the *B* factors of Trp533 and surrounding residues in *Nv*FdhAB. In all segments, the W active site is shown as sticks and spheres. The peptide chain is coloured by *B* factor (*B* factors were normalized for each structure independently), and 2*mF*_o_ − *DF*_c_ electron-density maps, at 1.0 r.m.s.d., are shown as a violet mesh. (*a*) The SSX_FdhAB structure. (*b*) The Fdh_CryoSamePrep FdhAB structure.

Lastly, conformational changes could also be observed for residues Phe160/Trp533 and for Met209 (Figs. 1 ▸*d* and 1 ▸*e*). These are currently not believed to play any specific role in catalysis. However, Trp533 becomes markedly disordered, with clear density only visible for the imidazole ring of the side chain (Fig. 4 ▸*a*). This is surprising, as the tryptophan is embedded in a hydrophobic pocket that includes the pyranopterin ring of MGD2. To exclude the possibility of an unexpected Trp→His or Trp→Phe mutation, large single crystals from the same protein-preparation batch were grown and a single-crystal dataset was recorded to 1.95 Å resolution at 100 K (Fdh_CryoSamePrep). Here, well defined electron density is observed for Trp533, indicating that no mutation has occurred (Fig. 4 ▸*b*). However, also in this structure the density of the benzene ring of the tryptophan is weaker than for the imidazole ring (to a lesser degree than in the room-temperature structure), which clearly provides further evidence that the side chain is mobile. This unusual behaviour of a buried tryptophan suggests that it may play a previously unsuspected functional role and this will be explored in future studies. Furthermore, comparing the substrate pocket of CryoSamePrep with Fdh_Cryo (PDB entry 6sdr) we notice that some features were not modelled in the CryoSamePrep structure (*e.g.* W1 and a second glycerol molecule close to His193) due to a less clear electron density for these data (Supplementary Figs. S4 and S5).

### 3.3. Radiation damage in the W active site and geometry comparison

**Figure 5:**
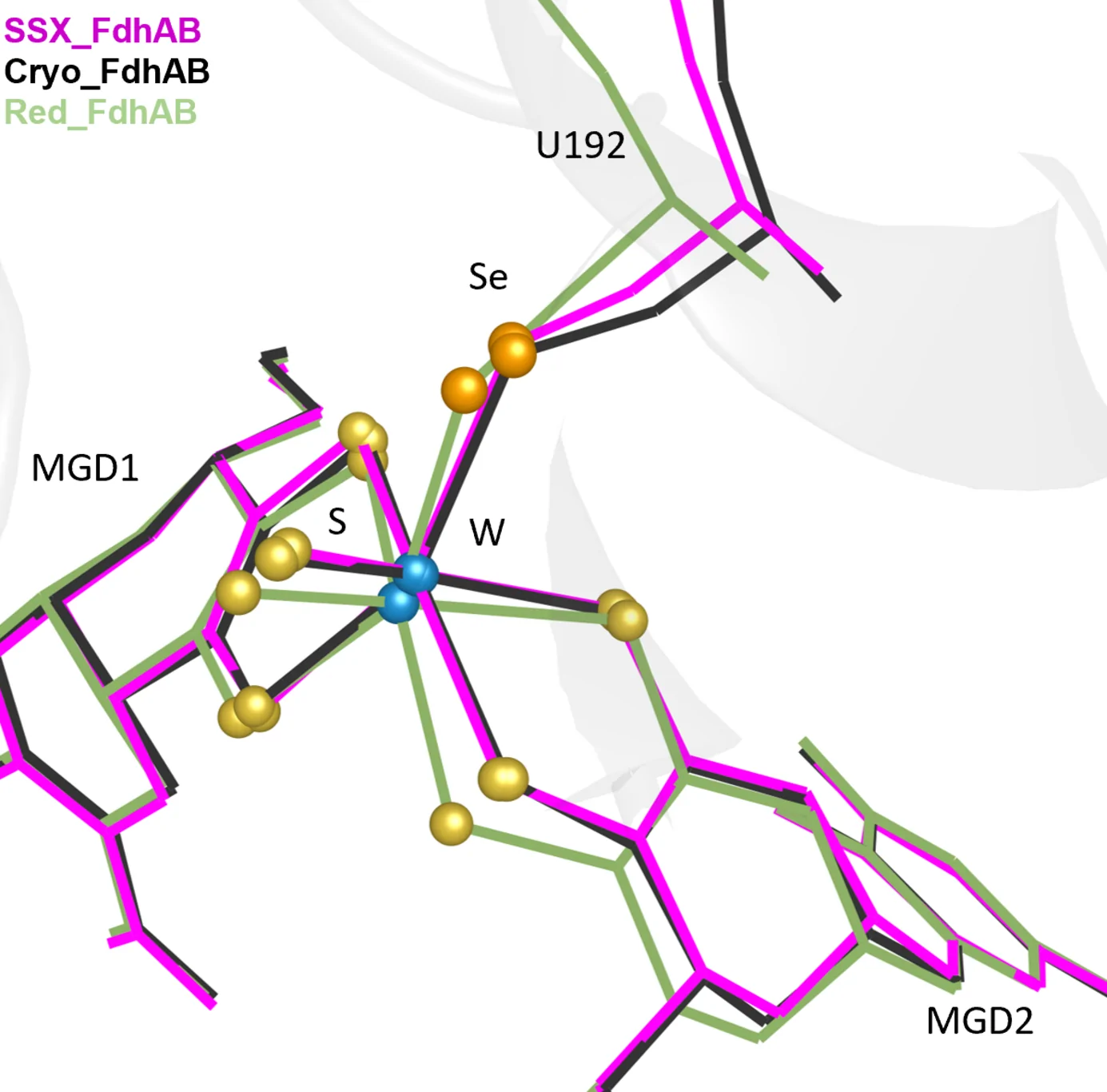
Superposition of the SSX (violet), the cryo *Nv*FdhAB (black; PDB entry 6sdr) and cryo formate-reduced *Nv*FdhAB (green; PDB entry 6sdv) active sites, aligned based on MGD1. The W site with the two MGD cofactors, the sulfido ligand and Sec192 is shown as sticks; the W ion, sulfido group and Sec192 Se atom are shown as spheres (light blue, yellow and orange, respectively).

The serial synchrotron data collection allowed us to obtain a low radiation-dose structure of *Nv*FdhAB with an average dose (exposed region) of 30 kGy, as estimated using the *RADDOSE*-3*D* online server (input data available in Supplementary Data S1; Dickerson *et al.*, 2024 ▸). Comparison of the W active site observed in the SSX structure with both the putative oxidized-state cryo structure (PDB entry 6sdr; average diffraction-weighted dose 2.2 MGy) and the cryo structure reported here (average diffraction-weighted dose 1.4 MGy; Zeldin *et al.*, 2013 ▸) shows that they are highly similar (Fig. 5 ▸), with closely matched bond distances, twisting and folding angles defined by the two pterin dithiolenes (Table 2 ▸), as proposed by Liu *et al.* (2021 ▸). In contrast, the previously published formate-reduced structure (PDB entry 6sdv; Oliveira *et al.*, 2020 ▸) shows an altered active-site geometry. The match between the active-site geometry observed in the low-dose serial data set and the putative oxidized-state cryo structures supports their assignment as the oxidized state, based on the structural data obtained. To fully ascertain this issue additional experiments, namely leveraging spectroscopic techniques, will be required.

## 4. Conclusion

*Nv*FdhAB microcrystals were produced and optimized for SSX data collection, yielding a low-dose, room-temperature SSX structure of the as-isolated protein at 1.93 Å resolution. Comparison with the published cryo as-isolated structure *Nv*FdhAB (PDB entry 6sdr) and the additional cryo structure reported here reveals a high degree of structural similarity. Notable is the conservation of the overall structural details of the recently reported retention site, despite the absence of the cryoprotectant glycerol that has been observed in this pocket in all cryo structures (Vilela-Alves *et al.*, 2026 ▸). Conformational changes in several residues that have been linked to the enzyme mechanism were observed, including Arg441, Thr196-Val197 and Gln409, as well as changes in water structure. Interestingly, a buried tryptophan residue (Trp533) close to the active site exhibited high mobility, suggesting that it may also have a functional role. Comparison of the geometry of the W active site in the low-dose serial structure with previously published cryo structures supports their assignment to the oxidized state, while further spectroscopic studies will be needed to fully clarify this question.

Overall, the new conformations of residues known to be involved in catalysis and regulation observed in this room-temperature structure highlight the importance of multi-temperature studies for understanding enzyme mechanism. The high-quality serial structure that is reported here enables future time-resolved mechanistic studies targeting possible catalytic intermediates in order to fully understand the catalytic mechanism of this biotechnologically relevant protein.

## Supplementary Material

PDB reference: W-formate dehydrogenase from *Nitratidesulfovibrio vulgaris*, 30gj

PDB reference: 30gk

Supplementary Data, Supplementary Tables and Supplementary Figures. DOI: 10.1107/S2059798326006923/ai5019sup1.pdf

## Figures and Tables

**Table 1:** Data-collection and processing statistics Values in parentheses are for the outer shell.

	Fdh_SSX	Fdh_CryoSamePrep
PDB code	30gj	30gk
Diffraction source	P14-2 T-REXX, PETRA III	P14, PETRA III
Wavelength (Å)	0.9762	0.9763
Temperature (K)	293	100
Dose	30 kGy	1.4 MGy
Crystal-to-detector distance (mm)	120.00	254.12
Rotation range per image (°)	0.00	0.10
Space group	*P*2_1_2_1_2_1_	*P*2_1_2_1_2_1_
*a*, *b*, *c* (Å)	65.5, 129.6, 151.9	64.9, 127.9, 149.4
Resolution range (Å)	98.79–1.93 (2.01–1.93)	59.50–1.95 (1.99–1.95)
No. of indexed lattices	11144	N/A
Total No. of reflections	7512081 (509525)	1177691 (59690)
No. of unique reflections	102629 (10129)	89865 (4440)
Completeness (%)	100.00 (100.00)	98.8 (98.6)
Multiplicity	73.2 (50.3)	13.1 (13.4)
〈*I*/σ(*I*)〉	3.0 (1.0)	18.8 (2.1)
*R*_split_/*R*_meas_†	0.285 (1.081)	0.096 (1.284)
Wilson *B* factor (Å^2^)	20.8	29.4
CC_1/2_	0.915 (0.377)	0.999 (0.739)
Final *R*_work_	0.185 (0.310)	0.212 (0.300)
Final *R*_free_	0.222 (0.329)	0.251 (0.324)
No. of non-H atoms		
Total	9663	9954
Protein	9220	9236
Ligand	132	225
Ion	2	2
Water	309	491
R.m.s. deviations		
Bond lengths (Å)	0.008	0.003
Angles (°)	1.504	1.212
Average *B* factors (Å^2^)		
Overall	27.87	39.83
Protein	29.61	42.57
Ligand	18.19	40.03
Ion	19.09	29.69
Water	28.02	40.43
Ramachandran plot		
Most favoured (%)	96.75	96.84
Outliers (%)	0.26	0.17
*MolProbity* score	1.17	1.40
Clashscore	1.95	3.00

† *R*_split._ is reported for serial datasets (*e.g.* Fdh_SSX), while *R*_meas_ is reported for cryo rotational datasets (*e.g.* Fdh_CryoSamePrep).

**Table 2:** Geometric parameters (angles and distances) of the W coordination shell in the SSX and cryo structures

	SSX *Nv*FdhAB structure (this work)	Cryo *Nv*FdhAB structure (PDB entry 6sdr)	Cryo *Nv*FdhAB structure from the same protein preparation as the SSX dataset (this work)	Cryo *Nv*FdhAB formate-reduced structure (PDB entry 6sdv)
Twisting angle† (°)	38.6	35.6	35.9	22.0
Folding angle† (°)	64.1	62.5	64.3	58.7
W–Se distance (Å)	2.6	2.5	2.5	2.5
W–S distance (Å)	2.2	2.3	2.4	2.5
W–S12_MGD1_ distance (Å)	2.5	2.4	2.4	2.4
W–S13_MGD1_ distance (Å)	2.4	2.4	2.4	2.3
W–S12_MGD2_ distance (Å)	2.5	2.5	2.5	2.4
W–S13_MGD2_ distance (Å)	2.4	2.5	2.4	2.5

† Dihedral angles defined by the two pterin dithiolenes (Liu *et al.*, 2021 ▸).
