# Supplementary material for: Room-temperature crystal structure of a metal-dependent W-formate dehydrogenase by serial synchrotron crystallography
Source: Acta Crystallogr D Struct Biol. 2026 Aug 10;82(Pt 9):1072–81. doi: 10.1107/S2059798326006923 (PMC13532692; doi:10.1107/S2059798326006923)
Supplement: Supplementary file 1 [file d-82-01072-sup1.pdf]

STRUCTURAL BIOLOGY

Volume 82 (2026)

Supporting information for article:

Room-temperature crystal structure of a metal-dependent W-formate dehydrogenase by serial synchrotron crystallography

Guilherme Vilela-Alves, Guilherme Martins, David von Stetten, Pedram Mehrabi, Inês A. C. Pereira, Maria João Romão, Arwen R. Pearson and Cristiano Mota

Suplementary data 1

Input used in RADDOSE-3D to obtain the dose estimates for the SSX-FdhAB dataset.

```
##############################################################################
#                         Crystal Block                       #
##############################################################################

Crystal

Type Cuboid
     # Crystal shape can be Cuboid or Spherical

Dimensions 30 30 6
     # Dimensions of the crystal in X,Y,Z in µm.
     # Z is the beam axis, Y the rotation axis and
     # X completes the right handed set
     #   (vertical if starting face-on).

PixelsPerMicron 2.0
     # This defines the coarseness of the simulation
     # (i.e. how many voxels the crystal is divided into.)
     # Preferably set as high as possible, however for a higher
     # value the simulation will take longer to complete.
     # Recommended to try increasing between 0.5 and 5 and ensure
     # the reported dose value converges as PixelsPerMicron increases.
     # As a rule of thumb, this needs to be at least 10x the beam
     # FWHM for a Gaussian beam.
     # e.g. 20µm FWHM beam -> 2µm voxels -> 0.5 voxels/µm

# NOTE: Use AngleP/AngleL if your crystal is not face-on to the beam.
# See RD3D user guide for more details

# Also need to specify the crystal composition below (Example case for insulin given):
AbsCoefCalc  RD3D
     # Absorption Coefficients calculated
     # using RADDOSE-3D (Zeldin et al. 2013).

UnitCell   65.50  129.60  151.90
     # unit cell size: a, b, c with alpha, beta and gamma angles default to 90°

NumMonomers  4
     # number of monomers in unit cell

NumResidues  1200
     # number of residues per monomer

ProteinHeavyAtoms W 1 Se 1 Fe 16 S 78
     # heavy atoms added to protein part of the
     # monomer, i.e. S, coordinated metals, Se in Se-Met
```

```
SolventHeavyConc Cl 1000
	# concentration of elements in the solvent
	# in mmol/l. Oxygen and lighter elements
	# should not be specified

SolventFraction 0.485
	# fraction of the unit cell occupied by solvent

###################################################################################
#                          Beam Block                          #
###################################################################################

Beam

Type Gaussian
	# beam profile can be Gaussian or TopHat
Flux 1.1e12
	# in photons per second (2e12 = 2 * 10^12)
FWHM 10 7
	# in µm, horizontal by vertical for a Gaussian beam
Energy 12.7
	# photon energy in keV

Collimation Rectangular 10 10
	# Horizontal/Vertical collimation of the beam
	# For 'uncollimated' Gaussians, 3xFWHM recommended


###################################################################################
#                          Wedge Block                          #
###################################################################################

Wedge 0 0
	# Start and End rotational angle of the crystal with Start < End

ExposureTime 0.005
	# Total time for entire angular range

# AngularResolution 0
	# Only change from the defaults when using very
	# small wedges, e.g 5°.

# NOTE: To define more complex geometries (helical, de-centred, or offset),
# see the StartOffset, TranslatePerDegree, and RotAxBeamOffset keywords
# in the User Guide
```

Input used in RADDOSE-3D to obtain the dose estimates for the Cryo-FdhAB dataset.

```
Job Definition (Input File)
##########################################################################################
#                              Crystal Block                              #
##########################################################################################

Crystal

Type Cuboid
    # Crystal shape can be Cuboid or Spherical

Dimensions 200 300 15
    # Dimensions of the crystal in X,Y,Z in µm.
    # Z is the beam axis, Y the rotation axis and
    # X completes the right handed set
    #   (vertical if starting face-on).

PixelsPerMicron 0.5
    # This defines the coarseness of the simulation
    # (i.e. how many voxels the crystal is divided into.)
    # Preferably set as high as possible, however for a higher
    # value the simulation will take longer to complete.
    # Recommended to try increasing between 0.5 and 5 and ensure
    # the reported dose value converges as PixelsPerMicron increases.
    # As a rule of thumb, this needs to be at least 10x the beam
    # FWHM for a Gaussian beam.
    # e.g. 20µm FWHM beam -> 2µm voxels -> 0.5 voxels/µm

# NOTE: Use AngleP/AngleL if your crystal is not face-on to the beam.
# See RD3D user guide for more details

# Also need to specify the crystal composition below (Example case for insulin given):
AbsCoefCalc  RD3D
    # Absorption Coefficients calculated
    # using RADDOSE-3D (Zeldin et al. 2013).

UnitCell   64.6 127.7 148.2
    # unit cell size: a, b, c with alpha, beta and gamma angles default to 90°

NumMonomers  4
    # number of monomers in unit cell

NumResidues  1200
    # number of residues per monomer

ProteinHeavyAtoms W 1 Se 1 Fe 16 S 78
    # heavy atoms added to protein part of the
    # monomer, i.e. S, coordinated metals, Se in Se-Met

SolventHeavyConc Cl 1000
    # concentration of elements in the solvent
    # in mmol/l. Oxygen and lighter elements
    # should not be specified
```

```
SolventFraction 0.485
      # fraction of the unit cell occupied by solvent

######################################################################################
#                              Beam Block                              #
######################################################################################

Beam

Type Tophat
      # beam profile can be Gaussian or TopHat
Flux 2.2e13
      # in photons per second (2e12 = 2 * 10^12)
FWHM 272 453
      # in µm, horizontal by vertical for a Gaussian beam
Energy 12.7
      # photon energy in keV

Collimation Rectangular 272 453
      # Horizontal/Vertical collimation of the beam
      # For 'uncollimated' Gaussians, 3xFWHM recommended


######################################################################################
#                              Wedge Block                              #
######################################################################################

Wedge 0 360
      # Start and End rotational angle of the crystal with Start < End

ExposureTime 27
      # Total time for entire angular range

# AngularResolution 2
      # Only change from the defaults when using very
      # small wedges, e.g 5°.

# NOTE: To define more complex geometries (helical, de-centred, or offset),
# see the StartOffset, TranslatePerDegree, and RotAxBeamOffset keywords
# in the User Guide
```

Table S1 Macromolecule production information

Source organism	*Nitratidesulfovibrio vulgaris* Hildenborough
DNA source	Mutated native DNA sequences
Expression vector	pMO9075
Plasmid construction method	Sequence and Ligation Independent Cloning-SLIC
Forward primer (Cloning)	*Nv*FdhA- 5'GCAGTCCCAGGAGGTACCATATGACAGTCACACGCAGACATTTCC3'<br>*Nv*FdhB- 5'CGCCTTCTTGACGAGTTCTTCTGAGGAGGTAGGTCATGGGAAAGATGTTCTTC3'
Reverse primer (Cloning)	*Nv*FdhA- 5'GAAACGATCCTCATCCCTATTTTTCGAACTGCGGGTGGCTCCAGACCTTGCGGACGTCCACCATG3'<br>*Nv*FdhB- 5'CTTCATGATCGGGTCTTTTCGTTCAGGCACGTGGCCGGAACAA3'
Expression host	*Nitratidesulfovibrio vulgaris* Hildenborough *ΔfdhAB* deletion strain
Complete amino-acid sequence of the protein produced	*Nv*FdhA-<br>MTVTRRHFLKLSAGAAVAGAFTGLGLSLAPTVARAELQKLQWAKQTTSICCYCAVGCGLIVHTAKDGQGRAVNV<br>EGDPDHPINEGSLCPKGASIFQLGENDQRGTQPLYRAPFSDTWKPVTWDFALTEIAKRIKKTRDASFTEKNAAGDLV<br>NRTEAIASFGSAAMDNEECWAYGNILRSLGLVYIEHQARIUHSPTVPALAESFGRGAMTNHWNDLANSDCILIMGS<br>NAAENHPIAFKWVLRAKDKGATLIHVDPRFTRTSARCDVYAPIRSGADIPFLGGLIKYILDNKLYFTDYVREYTNAS<br>LIVGEKFSFKDGLFSGYDAANKKYDKSMWAFELDANGVPKRDPALKHPRCVINLLKKHYERYNLDKVAAITGTSK<br>EQLQQVYKAYAATGKPDKAGTIMYAMGWTQHSVGVQNIRAMAMIQLLLGNIGVAGGGVNALRGESNVQGSTDQ<br>GLLAHIWPGYNPVPNSKAATLELYNAATPQSKDPMSVNWWQNRPKYVASYLKALYPDEEPAAAYDYLPRIDAGR<br>KLTDYFWLNIFEKMDKGEFKGLFAWGMNPACGGANANKNRKAMGKLEWLVNVNLFENETSSFWKGPGMNPAEI<br>GTEVFFLPCCVSIEKEGSVANSGRWMQWRYRGPKPYAETKPDGDIMLDMFKKVRELYAKEGGAYPAPIAKLNIAD<br>WEEHNEFSPTKVAKLMNGYFLKDTEVGGKQFKKGQQVPSFAFLTADGSTCSGNWLHAGSFTDAGNLMARRDKTQ<br>TPEQARIGLFPNWSFCWPVNRRILYNRASVDKTGKPWNPAKAVIEWKDGKWVGDVVDGGGDPGTKHPFIMQTHG<br>FGALYGPGREEGPFPEHYEPLECPVSKNPFSKQLHNPVAFQIEGEKKAVCDPRYPFIGTTYRVTEHWQTGLMTRRCA<br>WLVEAEPQIFCEISKELAKLRGIGNGDTVKVSSLRGALEAVAIVTERIRPFKIEGVDVHMVGLPWHYGWMVPKNGG<br>DTANLLTPSAGDPNTGIPETKAFMVDVRKVWSHPQFEK<br><br>*Nv*FdhB-<br>MGKMFFVDLSRCTACRGCQIACKQWKNLPAEETRNTGSHQNPPDLSYVTLKTVRFTEKSRKGPGIDWLFFPEQCRH<br>CVEPPCKGQADVDLEGAVVKDETTGAVLFTELTAKVDGESVRSACPYDIPRIDPVTKRLSKCDMCNDRVQNGLLP<br>ACVKTCPTGTMNFGDEQEMLALAEKRLAEVKKTYPGAVLGDPNDVRVVYLFTRDPKDFYEHAVA

Table S2 Summary of crystallization conditions

Dataset	SSX microcrystals	Cryo macrocrystals
Method	batch	vapor diffusion, hanging drop
Plate type	500 µL Microtube	24 well XRL plate Molecular Dimensions
Temperature (°C)	20 ºC	20 ºC
Composition of the protein solution	10 mg/ml *Nv*FdhAB, 20 mM Tris-HCl pH 7.6, 10% (*v/v*) glycerol and 10 mM $NaNO_3$	
Composition of reservoir solution	32 % (*w/v*) PEG 3350, 0.1 M Tris-HCl pH 8.0 and 1 M LiCl	22-26 % (*w/v*) PEG 3350, 0.1 M Tris-HCl pH 8.0 and 1 M LiCl
Composition of seed stock	*Nv*FdhAB WT microseeds, produced by harvesting macrocrystals of *Nv*FdhAB WT into 50 µL of reservoir solution and vortexing them with a plastic bead 10 times, 30 s on, 30 s off	
Volume and ratio of drop	30 µL in a ratio of 15:4.5:10.5 µL protein:microseeds:precipitant	2.2 µL in a ratio of 1:1:0.2 µL protein:precipitant:microseed dilution

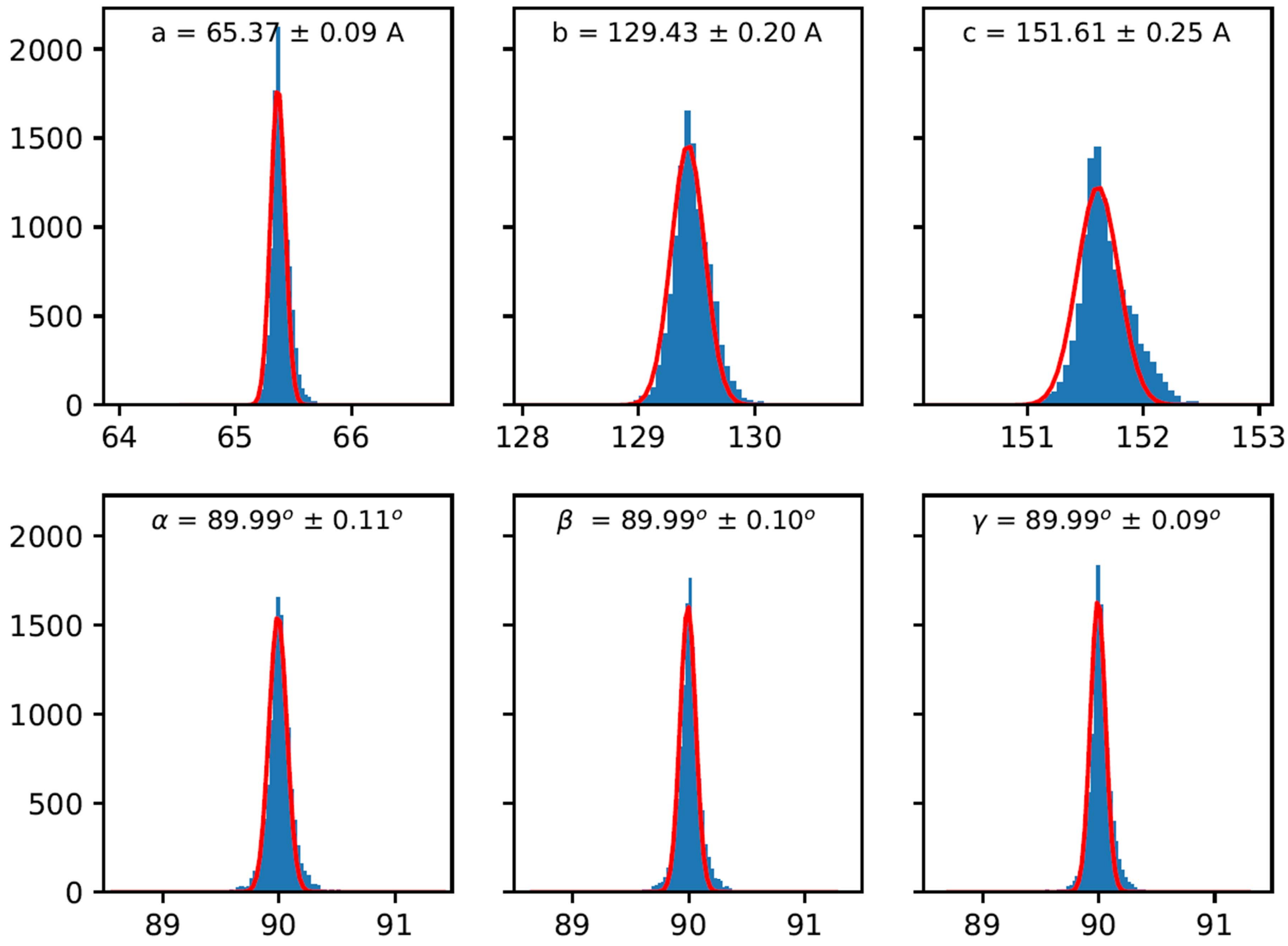

Figure S1 Histogram of the unit cell distribution for the room-temperature SSX FdhAB dataset.

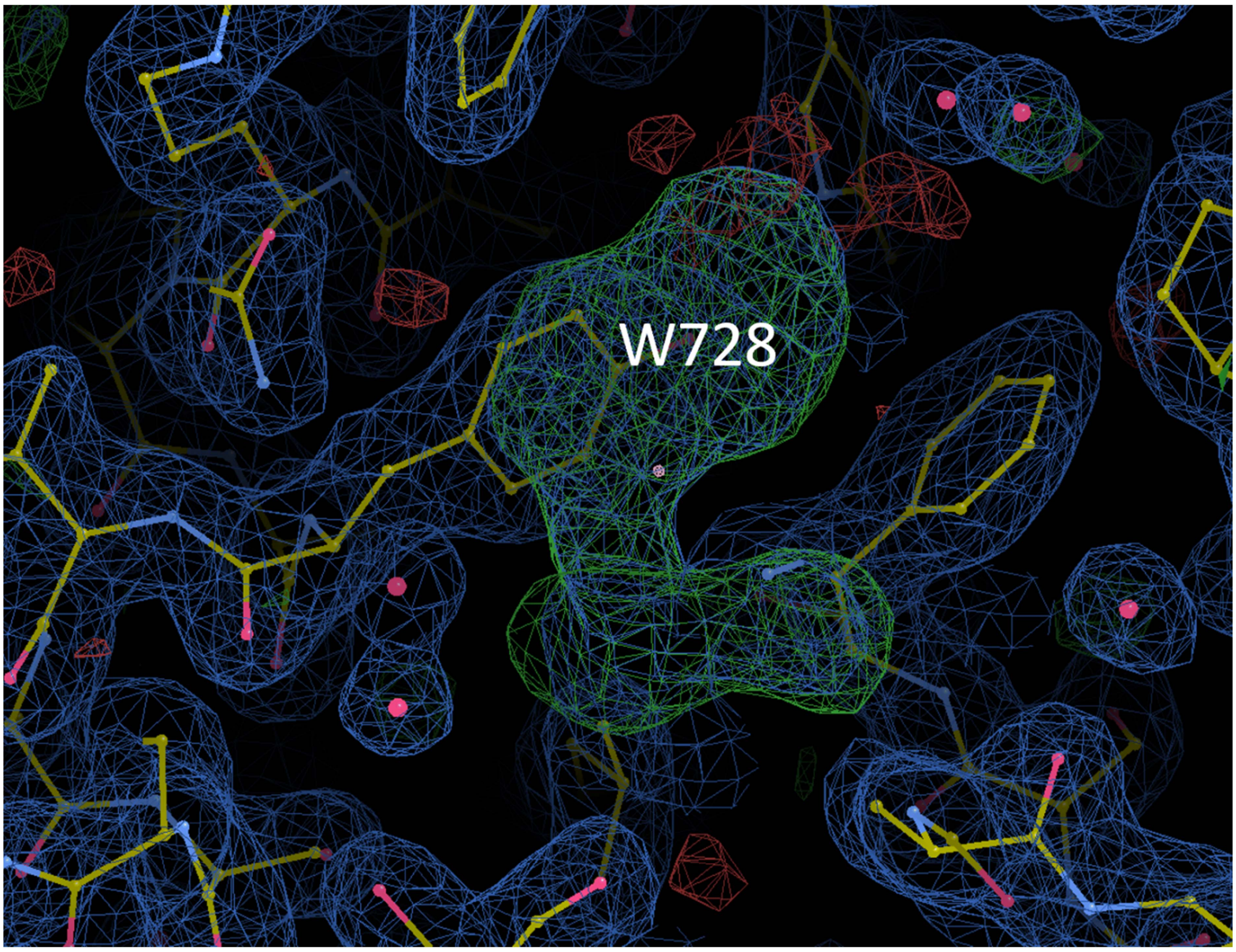

Figure S2 Omit map for W728 in the SSX *Nv*FdhAB structure. 2Fo-Fc and Fo-Fc electron density maps, respectively at 1σ and 3σ, are shown as blue and green/red meshes.

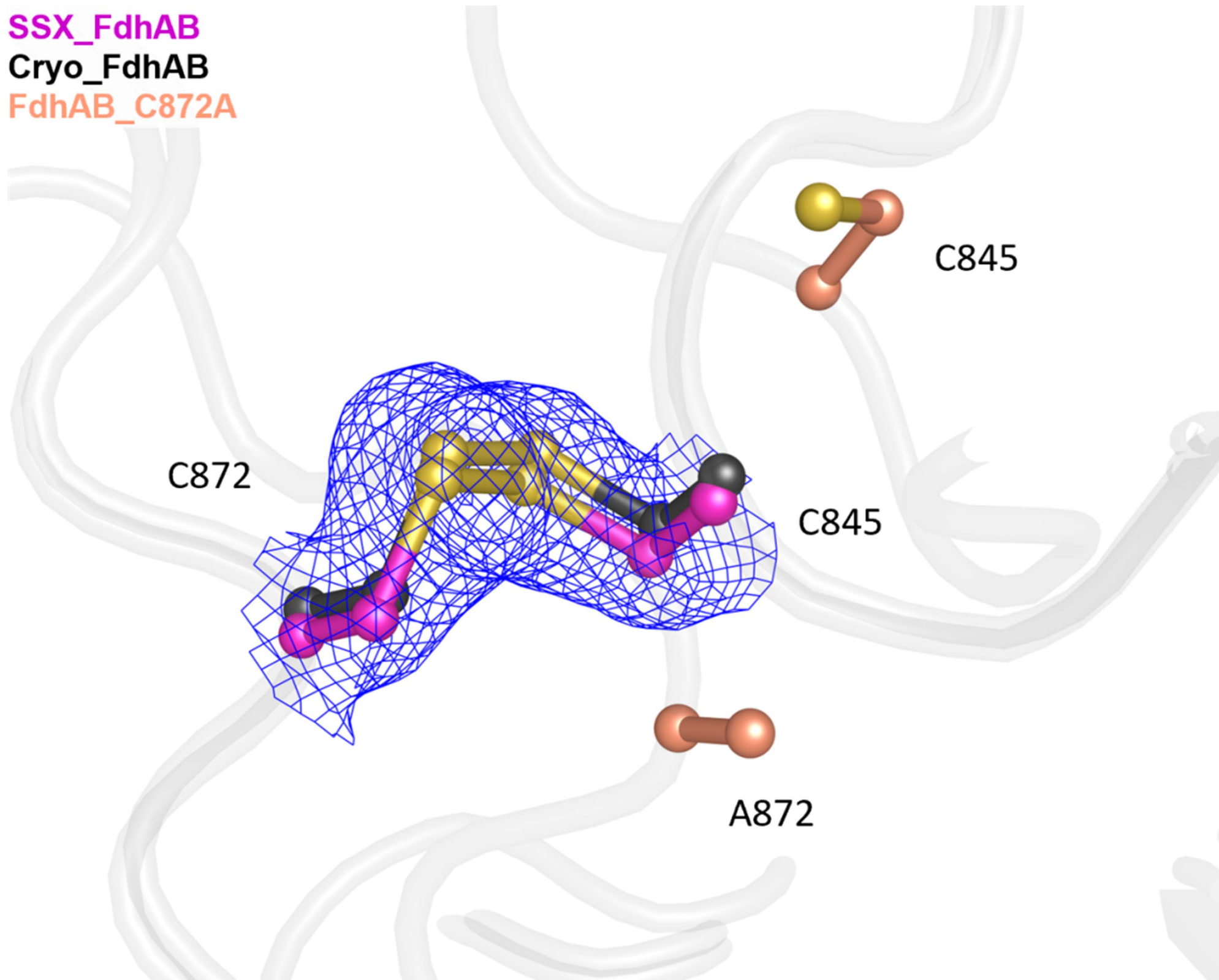

Figure S3 Superposition of the SSX, the cryo *Nv*FdhAB WT (PDB ID: 6SDR) and the C872A variant (PDB ID: 8CM6) *Nv*FdhAB structures, respectively in violet, black and orange. The solvent exposed C845-C872 disulfide bond is shown as well as the mutated C872A conformation for comparison. The 2Fo-Fc electron density map of the SSX dataset, at 1σ, is shown as a blue mesh.

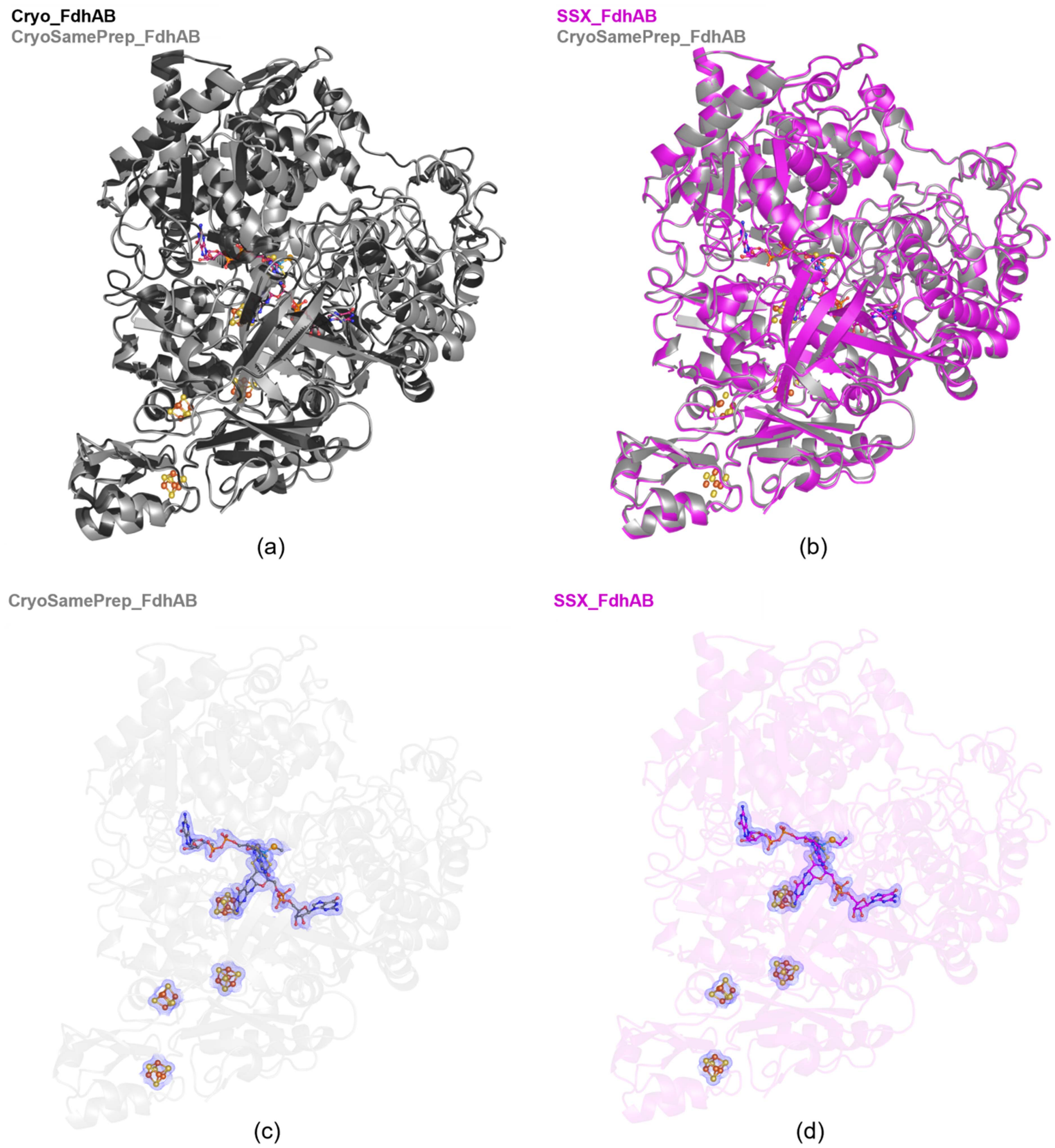

Figure S4 **(a)** Superposition of the Fdh_CryoSamePrep and Fdh_Cryo (PDB ID: 6SDR) structures, respectively in grey and black. **(b)** Superposition of the RT-SSX and Fdh_CryoSamePrep structures, respectively in violet and grey. **(c)** The W site with the two MGD co-factors and the four [4Fe-4S] clusters of Fdh_CryoSamePrep shown as sticks-and-balls, with their respective 2mFo-DFc electron density maps, shown as a blue mesh, at 1.0 rmsd. **(d)** The W site with the two MGD co-factors and the four [4Fe-4S] clusters of RT-SSX shown as sticks-and-balls, with their respective 2mFo-DFc electron density maps, shown as a blue mesh, at 1.0 rmsd.

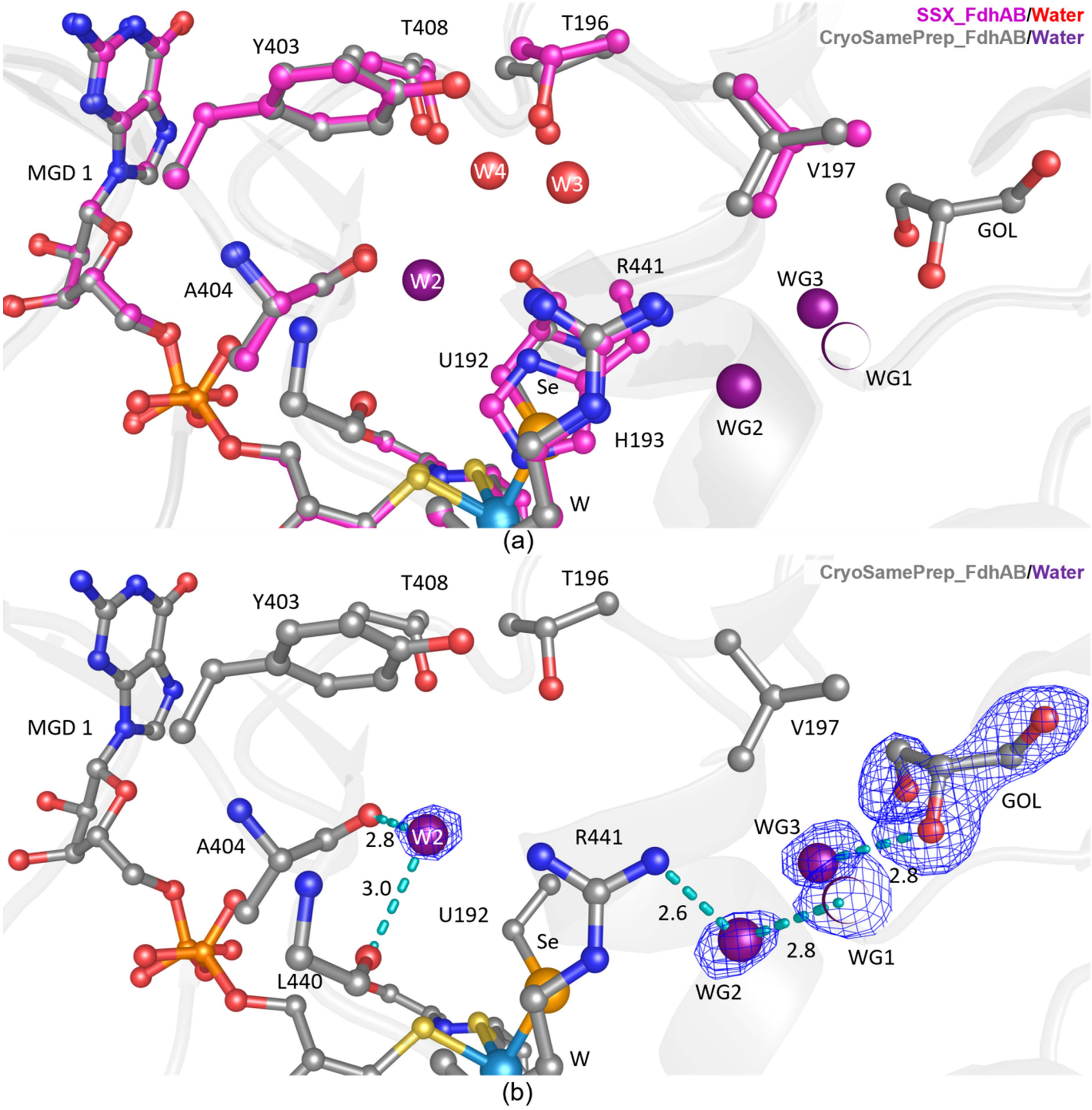

Figure S5 Differences in the vicinity of the retention site (Vilela-Alves et al., 2026)(Vilela-Alves et al., 2026) between the RT-SSX and Fdh_CryoSamePrep (PDB ID: 6SDR) structures, respectively in violet and grey. In all representations, the W active site is shown as sticks-and-balls; the W ion, sulfido group and U192 selenium atom are shown as spheres (light blue, yellow and orange respectively). 2*m*Fo-*D*Fc electron density maps are shown as a blue mesh, at 1.0 rmsd. Hydrogen bonds are shown as teal dashes. Distances are in Å. **(a)** Superposition of the RT-SSX and the Fdh_CryoSamePrep structures. The six water molecules are shown as spheres, in red and purple,

respectively, for the RT-SSX and the Fdh_CryoSamePrep structures. **(b)** The Fdh_CryoSamePrep structure.
